# Structure and contingency determine mutational hotspots for flower color evolution

**DOI:** 10.1002/evl3.212

**Published:** 2020-12-26

**Authors:** Lucas C. Wheeler, Boswell A. Wing, Stacey D. Smith

**Affiliations:** ^1^ Department of Ecology and Evolutionary Biology University of Colorado Boulder CO USA; ^2^ Department of Geological Sciences University of Colorado Boulder CO USA

**Keywords:** anthocyanin pathway, flavonoid, enzymes, evolutionary trajectories, pleiotropy, epistasis, simulations, complex phenotypes, genetic hotspots, predictability of evolution

## Abstract

Evolutionary genetic studies have uncovered abundant evidence for genomic hotspots of phenotypic evolution, as well as biased patterns of mutations at those loci. However, the theoretical basis for this concentration of particular types of mutations at particular loci remains largely unexplored. In addition, historical contingency is known to play a major role in evolutionary trajectories, but has not been reconciled with the existence of such hotspots. For example, do the appearance of hotspots and the fixation of different types of mutations at those loci depend on the starting state and/or on the nature and direction of selection? Here, we use a computational approach to examine these questions, focusing the anthocyanin pigmentation pathway, which has been extensively studied in the context of flower color transitions. We investigate two transitions that are common in nature, the transition from blue to purple pigmentation and from purple to red pigmentation. Both sets of simulated transitions occur with a small number of mutations at just four loci and show strikingly similar peaked shapes of evolutionary trajectories, with the mutations of the largest effect occurring early but not first. Nevertheless, the types of mutations (biochemical vs. regulatory) as well as their direction and magnitude are contingent on the particular transition. These simulated color transitions largely mirror findings from natural flower color transitions, which are known to occur via repeated changes at a few hotspot loci. Still, some types of mutations observed in our simulated color evolution are rarely observed in nature, suggesting that pleiotropic effects further limit the trajectories between color phenotypes. Overall, our results indicate that the branching structure of the pathway leads to a predictable concentration of evolutionary change at the hotspot loci, but the types of mutations at these loci and their order is contingent on the evolutionary context.

**Impact Summary**

A major topic of interest in evolutionary biology is determining to what degree evolution is repeatable and predictable, based on the characteristics of organisms and their environments. Numerous empirical studies have demonstrated that in many phenotypes evolutionary changes are achieved time and again using the same subset of genes in the underlying genetic pathways that control organism characteristics. Various hypotheses have been posed to explain this phenomenon. For example, many genes need to maintain roles in multiple functions, which can put strong constraints on which genes can be successfully mutated. Alternatively, it is thought that the structure of the genetic pathways themselves can restrict possible evolutionary paths. Here we examine the interaction between selection and pathway structure in a computational model of the anthocyanin biosynthetic pathway, which produces colorful pigments responsible for flower coloration. We simulate evolutionary transitions between different flower colors, analyze how the underlying pathway genes change, and determine how these changes depend on the starting and ending states. Our analyses demonstrate that the possible genes that can be altered to shift flower color are determined by a combination of the underlying pathway connections and the specific flower color transition under selection. These results indicate that the evolution of flower color is repeatable and predictable at the molecular level, given that sufficient information is known about the pathway structure and the direction of selection.

Evolutionary genetic hotspots are the repeated genetic loci of evolution (Stern and Orgogozo 2008; Martin and Orgogozo 2013), appearing across a variety of biological systems. Prominent examples include the roles of *MC1R* in animal melanism, *shavenbaby* in loss of *Drosophila* trichomes, and several loci of the anthocyanin biosynthesis pathway in floral coloration, all of which have repeatedly experienced mutations underlying phenotypic transitions (Kopp 2009). The repeated involvement of genetic hotspots has been used to argue for the predictability of evolution at the molecular level (Stern and Orgogozo 2008; Streisfeld et al. 2011). Although repeated events are simply a pattern of historical changes, they suggest there is something about hotspot loci that accounts for their over‐representation. For example, constraints imposed by the structure of pathways may restrict the accessible region of genotype‐phenotype space, favoring certain pathway targets and restricting the number of possible evolutionary paths (Vitkup et al. 2006; Morrison and Badyaev 2016). Such effects have been observed at the scale of protein evolution, where protein structure and function constrain the order and identity of possible amino acid substitutions (Weinreich et al. 2006; Bridgham et al. 2009; Franzosa and Xia 2009; Harms and Thornton 2014). Although we expect similar contingency in the evolution of organismal traits, with constraints on the order of changes at individual loci and across loci involved in phenotypic transitions (Edwards 2019), reconstructing such histories remains significantly more challenging.

Despite the many cases of genomic hotspots underlying phenotypic transitions (Martin and Orgogozo 2013), there are still major gaps in our understanding. First, the appearance of hotspots is likely closely tied to the structure of genetic pathways, but the precise relationship is not well defined. For example, the emergence of a hotspot in a situation where only one genetic mechanism exists is intuitively unsurprising (Shi and Yokoyama 2003), whereas the presence of hotspots in phenotypes that can be achieved via many possible mechanisms is more surprising (Ng and Smith 2016a, 2016b; Ahnert 2017). Second, it remains unclear whether the degree to which the concentration of changes at hotspot loci is due to the intrinsic genetic architecture of the trait or to the external selective forces that manifest as pleiotropic effects (Stern and Orgogozo 2008; Kopp 2009; Streisfeld and Rausher 2011; Wessinger and Rausher 2012). Finally, little is known about the importance of mutational order and the role of context dependence, as observed in protein evolution (Bridgham et al. 2009; Salverda et al. 2011; Gong et al. 2013; Harms and Thornton 2014; Shah et al. 2015; Kent and Green 2017; Starr et al. 2018). For instance, is the involvement of particular loci or types of mutations contingent on the starting state of the system (and thus on changes that occurred before)? Answering these questions will be essential for understanding the basis for repeated targeting of certain loci and the predictability of evolution.

Here, we model the evolution of a pathway about which we know a good deal from empirical work, allowing us to make direct comparisons between model predictions and observations from nature. The anthocyanin pathway, which is broadly conserved across flowering plants (Campanella et al. 2014), produces an array of colorful pigments falling into three classes: red pelargonidin‐derived pigments, purple cyanidin‐derived pigments, and blue delphinidin‐derived pigments (Fig. 1A). These pigments are responsible for most of the diversity in coloration across fruits and flowers; they are what make roses red and blueberries blue (Winkel‐Shirley 2001). Due to its deeply conserved topology, the anthocyanin pathway has become a prominent system for the study of genetic hotspots in phenotypic evolution (Kopp 2009; Streisfeld and Rausher 2011; Wessinger and Rausher 2012). The pathway is highly branched and reticulated, with multiple instances of competition between enzymes for substrates as well as competition between substrates for enzymes (Fig. 1A).

**Figure 1:**
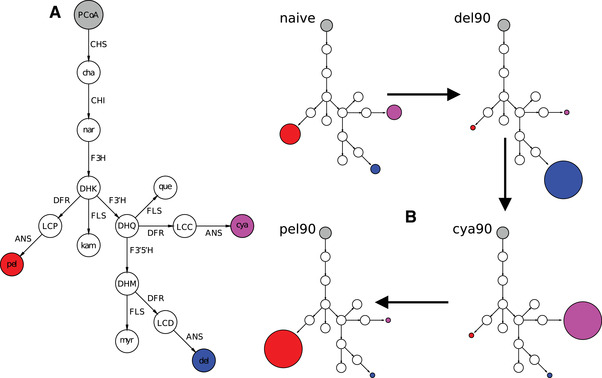
**Simulations of color evolution using an anthocyanin pathway model**. (A) The anthocyanin pathway, a portion of the larger flavonoid biosynthetic pathway (Winkel‐Shirley 2001), is shown with substrates at nodes and enzymes along arrows. Flux moves from PCoA at the top down through the branches of the pathway, terminating in the three types of pigments (pelargonidin (pel), cyanidin (cya), and delphinidin (del)), as well as the three flavonols kaempferol (kam), quercetin (que), and myricetin (myr). B) The simulations began with a naive state (all pathway parameters equal; see text) and moved first to blue (90% delphinidin), then to purple (90% cyanidin), and finally to red (90% pelargonidin). Substrates include: PCoA (P‐Coumaroyl‐CoA), cha (chalcone), nar (naringenin), DHK (dihydrokaempferol), DHQ (dihydroquercetin), DHM (dihydromyricetin), LCD (leucopelargonidin), LCC (leucocyanidin), LCD (leucodelphinidin). Enzyme abbreviations are: CHS (chalcone synthase), CHI (chalcone isomerase), F3H (flavanone‐3‐hydroxylase), F3'H (flavonol‐3'‐hydroxylase), F3'5'H (flavonoid‐3'5'‐hydroxylase), DFR (dihyroflavonol‐4‐reductase), FLS (flavonol synthase), ANS (anthocyanidin synthase).

Previously, we developed a biochemical model of the anthocyanin pathway, using a set of equations that describe flux through each pathway reaction. We then used this model to simulate evolution of the pathway between a “naive” state, wherein all enzyme concentrations and kinetic parameters started with identical values, and a “blue” phenotype where 90% of the total steady state pathway production was comprised by delphinidin (Wheeler and Smith 2019). Because of the structure of the pathway, where red pigments require the fewest steps (Fig. 1A), the naive state resulted in a phenotype of predominantly red pelargonidin pigmentation, and thus selection for blue pigmentation represents a shift to the farthest extreme in pathway output. With this experimental design, we found that simulated evolution involved the same genetic hotspots for color transitions as observed in nature. The involvement of these loci appeared related to their position in the pathway, which allows them to mediate internal trade‐offs between pathway products. This research, however, left many open questions, such as the relative importance of the pathway topology and the selection regime on evolutionary outcomes, the predictability of mutation order and mutation type, and the behavior of hotspot loci in more biologically realistic phenotypic transitions.

In this paper, we use our model to simulate the sequential evolution of two phenotypic transitions that are common in nature: from blue delphinidin pigments to purple cyanidin pigments and from that state to red pelargonidin pigments (Fig 1B). Considering the structure of the flavonoid pathway, these represent stepwise shifts from three to two to one hydroxyl groups on the flavonoid backbone, and phylogenetic modeling shows that these stepwise transitions are the primary mode of evolutionary change (Ng et al. 2018). Transitions from blue to purple and purple to red have occurred in parallel in many clades and have been studied at the genetic level in several species pairs (Wessinger and Rausher 2012). These analyses show that parallel evolution at the phenotypic level is commonly mirrored by parallelism in terms of genetic changes, often involving mutations of large‐effect (e.g., loss‐of‐function or down‐regulation) at a small subset of pathway loci. This repeated use of similar genetic changes has been related to pleiotropy, with fixed mutations being those assumed to carry fewer negative consequences (Streisfeld and Rausher 2011; Wessinger and Rausher 2012). However, it is also possible that the topology of the pathway creates internal constraints that bias evolution toward the observed fixed mutations (Clotault et al. 2012a; Morrison and Badyaev 2017).

Taking a computational approach, we explore the range of evolutionary trajectories connecting blue, purple, and red phenotypes, and ask how the mutations fixed during these trajectories compare to those observed in nature. In comparing these transitions, we also aim to address general questions about how selected mutations are expected to differ based on the nature of the selection on the pathway. These include (1) Does the location of the phenotypic optimum in pigmentation‐space affect the identities of hotspot loci? (2) Are the type, direction, and order of mutations in evolutionary trajectories predictable based on pathway topology and location of the optimum? (3) How does the control of pathway dynamics shift to accommodate new pigment phenotypes? By dissecting a large number of simulated evolutionary trajectories for a sequential set of phenotypic transitions, we are able to determine the importance of pathway structure, selective context, and changes to the pathway dynamics in altering the pigmentation phenotype. Our results identify the mechanisms of pathway evolution, generate testable predictions for future empirical studies, and provide a generally applicable framework for the emergence and behavior of hotspot loci based on interactions between pathway topology and selection.

## Methods

### DESIGN OF THE KINETIC MODEL OF THE ANTHOCYANIN PATHWAY

We constructed a kinetic model of the anthocyanin biosynthetic pathway that is designed to capture the most salient pathway features. The model consists of a set of differential equations (described below; also detailed in the Supporting Information) that capture the topology and dynamics of a simplified representation of the anthocyanin pathway. It includes the branches that produce the anthocyanidin pigments (pelargonidin, cyanidin, and delphinidin), as well as the competing set of branches that produce the flavonols (kaempferol, quercetin, and myricetin) from shared precursor compounds (Fig. 1A; Fig. S1). We incorporated several simplifying assumptions into the model, such as ignoring the linked production of flavone compounds (Winkel‐Shirley 2001) and the decision to exclude the overlap in activity between the F3'H and F3'5'H enzymes, because this shared activity is variable in nature (Kaltenbach et al. 1999; Seitz et al. 2007; Falginella et al. 2010).

To represent the pathway reactions, we used a generalized Michaelis‐Menten rate law formulation (Chou and Talaly 1977). We specified irreversible rate laws for each enzymatic reaction in the pathway model using the Tellurium library (Choi et al. 2018), as previously described (see Supplemental text and (Wheeler and Smith 2019)). Each enzyme rate law in the pathway has three different types of parameters: $K_{cat}$; the catalytic turnover rate, $K_{m}$; the Michaelis constant (related to a dissociation constant), and $E_{t}$; the enzyme concentration. This rate law formulation allows us to incorporate substrate competition for enzymes with multiple substrates, such as DFR, by using unique $K_{cat}$ and $K_{m}$ parameters for the different substrates (see Fig. 1A). We previously determined that irreversible rate laws were a reasonable approximation for the behavior of the anthocyanin pathway (Wheeler and Smith 2019) and a simple irreversible model has also been shown to fit well to the kinetic results of *in vivo* experiments 2013. An added advantage is that the irreversible model requires fewer parameters. To allow calculation of a steady state solution for the system of equations that represent the pathway dynamics, we instituted two boundary processes: (1) the incoming upstream concentration of PCoA (Fig. 1) is fixed at a constant value, (2) the rate of transport of the products out of the pathway system is determined by $K_{sink}$ (a rate constant shared by all final products of the pathway: pelargonidin, cyaninidin, delphinidin, kaempferol, quercetin, and myricetin; see Fig. 1) multiplied by the product concentration (see model specification in, e.g., Supporting Information File “cyanidin‐to‐pelargonidin‐simulations.py”).

### DESIGN SCHEME FOR THE EVOLUTIONARY SIMULATIONS

We simulated sequential phenotypic transitions (Fig. 1B) using the evolutionary algorithm implemented in our python package enzo (Wheeler and Smith 2019) (https://github.com/lcwheeler/enzo). Briefly, our algorithm works by sampling numerical mutations from a gamma distribution with α=0.8 and β=3, chosen so that the mutation process samples both large negative and positive changes in addition to smaller‐effect mutations. These mutations are multiplicative shifts that can result in either an increase or decrease to the value of a single $K_{cat}$, $K_{m}$, or $E_{t}$ parameter. For example, a large negative mutation that reduces the value of a $K_{cat}$ parameter to nearly zero will drastically reduce the activity of the enzyme on that applicable substrate, which can be thought of as a coding mutation resulting in a loss‐of‐function. Meanwhile, a large negative mutation that reduces an $E_{t}$ parameter to near zero could be thought of as a regulatory loss‐of‐expression mutation, which would affect all reactions catalyzed by the enzyme. Each iteration of the algorithm introduces a random mutation to a randomly selected parameter and then re‐calculates the steady state concentration of all chemical species in the pathway. Since the focus of this study is on shifts among pigments (as opposed to changes in the amount of pigmentation), we discarded mutations that altered total steady state production beyond a 10% tolerance. This experimental design is in line with empirical work, where species can shift between pigment types while keeping the total anthocyanin levels relatively constant (Berardi et al. 2016; Esfeld et al. 2018), resulting in flowers of different hues but similar color intensity. Natural and engineered systems also provide evidence of trade‐offs between anthocyanins and flavonols, suggesting constraints on total flavonoid content (Nielsen et al. 2002; Davies et al. 2003; Nakatsuka et al. 2007; Yuan et al. 2016). For mutations falling within the tolerance, a selection coefficient is calculated using the fitness function: $W=\exp(-{\left(ratio_{current}-ratio_{opt}\right)}^{2})$, which depends on the distance of the steady state ratio of a target pigment to the sum of all pigments ($ratio_{current}$) from a pre‐defined phenotypic optimum ($ratio_{opt}$) (Clark 1991; Wright and Rausher 2010; Rausher 2013; Wheeler and Smith 2019). A fixation probability is calculated based on a formula ($1-e^{-s}$) that is weighted by the selection coefficient. The mutation is then either fixed or discarded probabilistically. Neutral (s=0) and deleterious (s<0) mutations are discarded for efficiency, because their fixation probabilities are negligible. The algorithm performs a series of iterations until either the optimum is reached within a defined tolerance (here 10%) or a pre‐set maximum number of iterations (here 50,000) is reached.

Previously, we used this framework to study the transition from a “naive” pathway model (all kinetic parameters of a certain type are initialized with equal numerical values) to a state wherein 90% of the total steady state concentration of all chemical species was composed of the blue delphinidin pigment (see (Wheeler and Smith 2019)). Here, we focused on transitions between naturally occurring phenotypes (blue, purple and red flowers) (Fig. 1B). The initial starting state was the original naive pathway model mentioned above. We evolved the model from the naive phenotype to a state wherein delphinidin composed 90% of the total *anthocyanin* concentration at steady state (the “blue” phenotype), with a total of 10,000 unique simulated trajectories. We then calculated the mean evolved value for each model parameter and initialized a new starting state with these values. We evolved this mean 90% delphinidin state (hereafter referred to as simply the delphinidin state) to a 90% cyanidin state (corresponding to “purple”; hereafter referred to as simply the cyanidin state) and finally repeated the procedure to simulate the transition from cyanidin to 90% pelargonidin (corresponding to “red”; hereafter referred to as the pelargonidin state). Organizing the simulations around this stepwise sequence of transitions allowed us to circumvent issues that arise from epistasis in the pathway model, wherein different starting states can result in differences between trajectories that are difficult to interpret (Ng et al. 2018).

Finally, we implemented two additional simulation experiments to examine the effect of the branched pathway structure on the properties of evolutionary trajectories. First, we created a simplified linear pathway model (the sub‐pathway containing only the steps leading to pelargonidin production, initialized with the parameters from the naive model), and evolved this model toward an optimum defined by a threefold increase in steady‐state pelargonidin concentration relative to the starting state. Second, we imposed a constraint that the ratio of pelargonidin at steady state to all other pathway substrates/products remain constant. To examine the effect of the fitness function alone, we then applied this same scenario to the (branched) naive anthocyanin pathway model. We carried out 2,000 replicates of each of these simulations. See Supporting Information Supplemental Methods for details on the subsequent analyses of all simulations.

## Results

### EVOLUTION OF HOTSPOT ENZYMES IS DEPENDENT ON THE PHENOTYPIC TRANSITION

**Figure 2:**
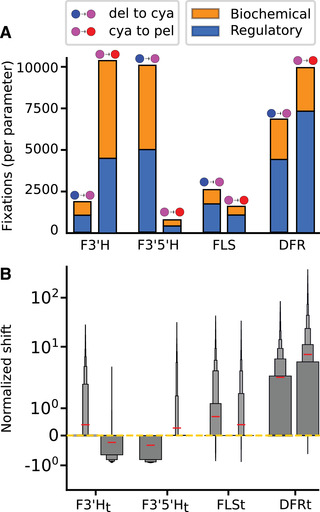
Hotspots vary depending on transition type with consistent patterns of mutation. The delphinidin to cyanidin and cyanidin to pelargonidin transitions are labeled (see Fig. 1B). (A) Overall proportions of biochemical and regulatory mutations for each hotspot locus in both phenotypic transitions, normalized by the total number of each parameter type per enzyme (e.g., DFR has three $K_{cat}$ values and three $K_{M}$ values for a total of six “biochemical” parameters). Stacked orange and blue boxes show the proportion of biochemical ($K_{M}$and $K_{cat}$) and regulatory ($E_{t}$) mutations (see legend). (B) Boxenplot (Hofmann et al. 2017) of normalized directional shifts for the concentration parameter from each of the four enzymes shown in panel (A). Box width is proportional to the number of data points in the enclosed region. The mean of each distribution is shown by horizontal red line. Directional shifts are shown on a log scale. Distributions of directional shifts for the $K_{M}$, $K_{cat}$, and $E_{t}$ for all the enzymes in the pathway are shown in Fig. S6.

Although the two trajectories were of similar length and overlapped in the loci targeted, we observed marked differences in the type and frequency of mutations at pathway enzymes. The median length for trajectories in both phenotypic transitions is four steps (see trajectory length distributions in Fig. S2). Roughly 99% of all fixed mutations in both transitions are spread across four loci, F3'5'H, F3'H, DFR, and FLS (Fig. S3), which we identified as pathway hotspots in our earlier study (Wheeler and Smith 2019). However, the frequencies of fixation at these hotspot enzymes depend on the phenotypic transition under selection (Fig. 2A). The predominant switch is in the fixation frequencies of mutations to F3'5'H and F3'H. In the transition from blue delphinidin to purple cyanidin, mutations at F3'5'H are fixed much more frequently than those at F3'H. This pattern is reversed in the transition from purple cyanidin to red pelargonidin, where fixed mutations to F3'5'H parameters are relatively rare (Fig. 2A). In comparison, the fixation frequency of mutations at DFR and FLS are quite similar in both transitions. These results demonstrate the dependence of hotspot behavior on the axis of variation under selection.

We found that there is an overall bias toward regulatory mutations (enzyme concentration), compared with biochemical mutations (changes to kinetic parameters), in both phenotypic transitions. Regulatory mutations represent >50% of all fixations at the four hotspot enzymes. Nonetheless, both biochemical and regulatory mutations are highly represented in the trajectories of both transitions (Fig. 2A). Changes in concentration of F3'H and F3'5'H can easily drive shifts in the relative production of downstream products due to their location at branch points that commit flux toward one or another pigment branch (Fig. 1A). However, the reason for the effects of regulatory changes at DFR and FLS is less immediately obvious. We previously observed that the naive pathway has an inherent bias toward pelargonidin/kaempferol production, due simply to the early partitioning of flux down these committed branches. Thus, a shift in DFR concentration affects the steady state production along each branch differently (Fig. S4). These differences can be accentuated or suppressed by differential biochemical changes to DFR activity on pigment precursors (see Supporting Information and Fig. S5 for a detailed explanation of this general phenomenon). Likewise, the ability of FLS to draw flux away from all anthocyanin pigment branches allows it to redirect flux to tune the anthocyanin output in a manner that is also dependent on the relative efficiency of the competing reactions.

We found that the phenotypic transitions are characterized by predictable sets of positive and negative mutations at hotspot enzymes. Specifically, certain parameters at the hotspot loci are shifted in a way that increases the activity of a particular reaction, while others reduce the activity. The distributions of these shifts for the $E_{t}$ concentration parameters are shown in Fig. 2B (see Fig. S6 for the shift distributions of all $E_{t}$, $K_{cat}$, and $K_{M}$ parameters). As with the fixation frequencies at each enzyme, these directional changes are also contingent on which phenotypic transition is being made (Fig. 2B, Fig. S6). The most striking example can be seen in the shift distributions for concentration parameters of F3'H and F3'5'H in Fig. 2B. In the transition from the delphinidin to the cyanidin, fixed mutations to F3'H $E_{t}$ are almost exclusively positive, typically representing a several‐fold increase in F3'H concentration. Meanwhile mutations to F3'5'H concentration are negative, resulting in a several‐fold decrease. This pattern is exactly reversed in the transition from cyanidin to pelargonidin (Fig. 2B). Combined with mutations to $K_{cat}$ and $K_{M}$ (Fig. S6), the changes in F3'H and F3'5'H concentration resulted in large shifts in the activity of these enzymes.

### TRAJECTORIES HAVE CONSISTENT STRUCTURE WITH BIASED ORDER OF FIXED MUTATIONS

**Figure 3:**
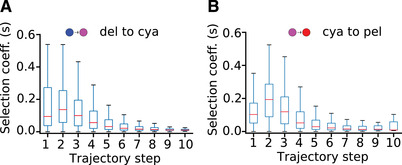
Largest fixed mutations occur at early (but not earliest) trajectory steps. Distributions of *fixed* selection coefficients at each trajectory step over all 10,000 simulations per phenotypic transition, shown as boxplots for (A) delphinidin to cyanidin and (B) cyanidin to pelargonidin. The curves are peaked around the second step, resembling a chemical activation barrier. Red lines indicate median values.

Looking across simulations, the curves for fixed mutations show a skewed distribution, with the largest mutations occurring in the early (but not earliest) steps (Fig. 3). There is a peak around the second trajectory step representing the largest selection coefficients (Fig. 3). This is despite the aggregate distributions of selection coefficients for all fixed mutations, regardless of trajectory step, following a roughly exponential distribution (see Fig. S7). It is also worth noting that during early development of the model we experimented with a uniform (rather than gamma) distribution for parameter mutation sizes, making very large effects on enzyme activity as probable as very small effects. This markedly different distribution did not qualitatively affect the stepwise distribution of fitness effects or the identities of common targets of fixation, indicating that the shape of evolutionary trajectories is not due to our choice for the form of the mutational spectrum. We also confirmed that the peaked pattern in the stepwise distributions was not an artifact of aggregating data from trajectories by ranking the selection coefficients of fixed mutations in each individual trajectory. In both the blue to purple and the purple to red transitions, we observed that in >50% of trajectories the selection coefficient of the mutation fixed in the second step was larger than that in the first step. This observation demonstrates that typical trajectories exhibit a pattern similar to that shown by the aggregated distribution curves in Fig. 3.

We hypothesized that the peaked shape of the selection coefficient curves could be due to the interplay between our chosen fitness function and the complex interactions within the pathway. These interactions could restrict the availability of largest‐effect mutations at the beginning of trajectories. As described in the Methods section, we tested this idea by employing two additional simulation experiments. A simple linear pathway model (containing only the series of reactions leading to pelargonidin), evolved under selection for increased absolute pelargonidin production with fixed ratio of pelargonidin to the other species (see Methods), exhibited a roughly exponential distribution of selection coefficients over trajectory steps (Fig. S8). This result suggests that the branching structure of our main model could contribute to the peaked stepwise distributions of selection coefficients during adaptive walks. However, when we applied the same fitness function to the naive pathway model (with the full branching structure), we observed a similar exponential distribution of selection coefficients (Fig. S9). This result indicates that the combination of directional selection on target‐pigment ratio and stabilizing selection on total pathway production (see Methods) is likely the main cause of the peaked shape of the adaptive walks. It is worth noting that the fitness function used in these additional simulations (see Methods) results in increased frequency of fixed mutations at both upstream and downstream pathway enzymes (Fig. S10). This observation is consistent with the imposed selection on overall increased concentration of an end‐product (requiring increased total pathway flux) rather than selection to maintain the total flux of the starting state.

**Figure 4:**
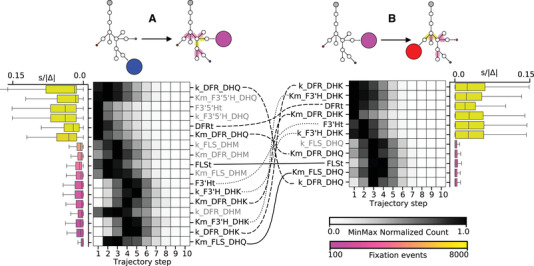
Order of fixed mutations strongly influenced by sensitivity. For each transition (delphinidin to cyanidin in A and cyanidin to pelargonidin in B), heatmaps show the distribution of fixation events across the trajectory steps for all parameters making up at least 1% of all fixed mutations. Parameter names are listed in descending order of total number of fixation events (colored pink to yellow). Boxes in the heatmap show the fixation events at each step in the trajectories across the 10,000 simulations; counts are normalized on a MinMax (0,1) scale for comparison (see MinMax Normalized Count scale bar). Boxplots show the *complete* distributions of “sensitivity” values (|s/Δ|; where s is the selection coefficient and Δ is the normalized directional mutation size
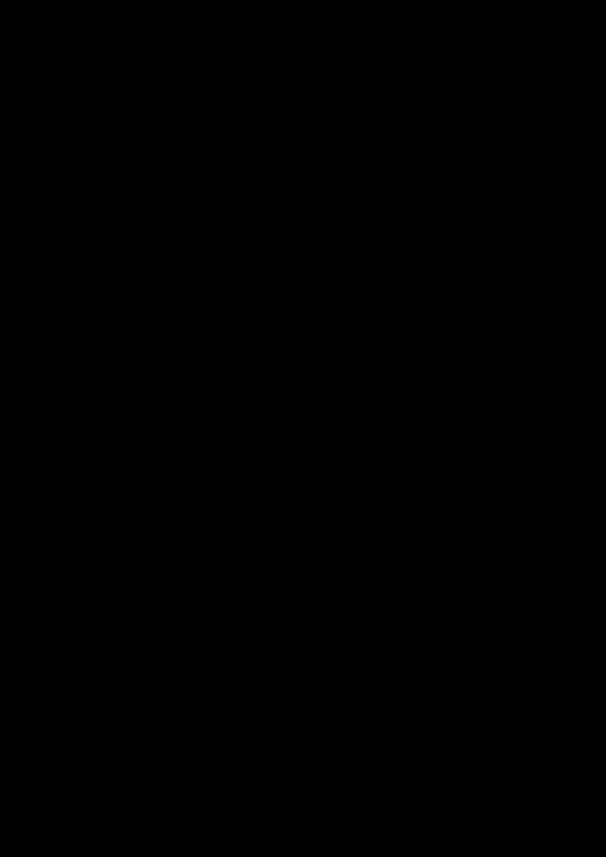
: (mutantvalue−previousvalue)/(previousvalue)) for *all* mutations for the given parameter (see Fig. S11 for more details on the sensitivity calculation). Reactions involving the more frequently targeted/higher‐sensitivity parameters (yellow) and less frequently targeted/lower‐sensitivity parameters (pink) are highlighted on the pathway diagrams at the top of figure. Parameter notation: $Km=K_{M}$(e.g., Km_DFR_DHQ), $k=K_{cat}$ (e.g. k_DFR_DHQ), and $Et=E_{t}$ (e.g., DFRt), where both the enzyme and the substrate associated with the kinetic parameters is indicated (e.g., k_DFR_**DHQ**). Parameters shared across transitions are connected by curved lines, where line style is matched for parameters of the same enzyme: DFR (dashed), F3'H (dotted), and FLS (solid). Parameters unique to each transition are written in gray.

We next examined the series of mutations underlying the two focal transitions. Despite relying on mutations at different parameters, both the blue to purple and purple to red transitions showed a similar tight relationship between the sensitivity of the pathway to particular mutations and the position of those mutations in the trajectories. Specifically, mutations at enzyme parameters with higher mean sensitivity (see Supplemental Methods) were biased toward earlier fixation in trajectories, as can be seen by the similarity in heat maps in Fig. 4. The result of higher sensitivity is that, for those parameters, a small mutation to the parameter value can induce a bigger shift in pathway function on average. Since there is a general trend for larger‐effect mutations to be fixed earlier in trajectories, these sensitivity differences manifest in certain parameters becoming concentrated among early mutations while others are skewed toward the middle or end of trajectories (Fig. 4). There is also a positive relationship between sensitivity and total number of fixed mutations at a given parameter (Fig. 4). We also noted that the parameters contributing to the cyanidin to pelargonidin transition are almost entirely a subset of those that contribute to the delphinidin to cyanidin transition, a pattern resulting from the effective clipping of the delphinidin branch during the first simulated transition. Nonetheless, the order of mutations at those parameters differs markedly between trajectories (dashed lines in Fig. 4), indicative of the corresponding differences in sensitivities. For example, the blue delphinidin state is highly sensitive to mutations in k_DFR_DHQ (the $K_{cat}$ for DFR on the DHQ precursor), making it an early target, while the purple cyanidin state has little sensitivity to mutations in that parameter, leaving them for later in the trajectories.

### CONTROL OF PATHWAY DYNAMICS IS RE‐ARRANGED TO ACHIEVE SWITCHES BETWEEN PIGMENT PHENOTYPES

**Figure 5:**
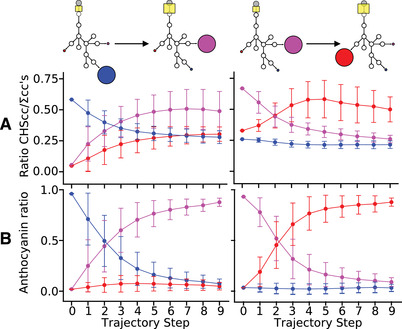
Pathway flux dynamics are re‐arranged to accommodate phenotypic transitions. Left column is delphinidin to cyanidin transition, right column is cyanidin to pelargonidin transition (see pathway diagrams at the top). (A) Trajectories through the concentration control coefficient (CC) space, depicting the absolute value of the ratio of the CC of the CHS enzyme for each pigment to the sum of all CC values for that species ($|CC_{target}^{CHS}/\sum CC_{target}^{all\;enzymes}|$) across all trajectory steps. The position of CHS in the pathway is highlighted by the yellow boxes in the pathway diagrams at the top, with the change in size denoting increased relative control over target pigment. The proportion of total control over the target species possessed by CHS increases to saturation in both cases. Inflection points of these curves align with inflection points of the pigment ratio curves in panel (B). The overshoot in the relative control of CHS over pelargonidin (red) during the transition from cyanidin (purple) to pelargonidin is likely due to the intrinsic bias of the pathway toward pelargonidin production. Error bars are one standard deviation. (B) Mean trajectories through pigment space (in relative amounts of each); the relative amount of the target pigment is proportional to fitness in our model. Colors follow above (delphinidin=blue, cyanidin=purple, pelargonidin=red). Error bars are the standard deviation of the mean.

The primary change to the pathway dynamics during each phenotypic transition is a re‐arrangement of the underlying structure of control that enzymes exert over steady state concentrations of chemical species in the pathway (see Supplemental Methods). The predominant shift in the pathway control structure is summarized in Fig. 5A. The pathway is effectively linearized down the path (series of reactions) leading to the target pigment, for example, cyanidin or pelargonidin. This re‐arrangement transforms the path to the target pigment into a path of reduced resistance. These shifts in pathway control are largely reproducible, falling within a fairly narrow range across the majority of simulations (Fig. 5A, Fig. S13).

The linearization effect is achieved by weakening the control that the branching enzymes (such as F3'5'H and F3'H; see Fig. S12) have over the target pigment, while strengthening the control they have over the off‐target species. Particularly for the other two non‐target anthocyanin pigments, the branching enzyme control coefficients become more negative, which can be interpreted as a stronger dampening of off‐target pigment production (Fig. S12). The net effect of these shifts is to grant the dominant control over flux down the target path (i.e., cyanidin or pelargonidin) to CHS, the furthest upstream enzyme in the pathway, which is itself negligible as a direct target of fixed mutations (Fig. 1A, Fig. 5A, Fig. S12). The trajectories of the absolute value of the ratio of the CHS concentration control coefficient for the target pigment to the sum of all coefficients for that pigment are shown in Fig. 5A. In both transitions this ratio converges to a stationary value over the course of simulations, mirroring the trajectories through pigment space shown in Fig. 5B.

The mean trajectories of the target pigment ratio (relative to the total anthocyanins produced at steady state) are the phenotype‐level manifestation of the underlying changes to the pathway control structure. They follow a sigmoidal curve across trajectory steps (Fig. 5B). Shifts in flux down the target branch result in trade‐offs with other branches that are particularly pronounced between the current target species and the target species from the previous transition in the sequence. This is due to the constraint on the total amount of material produced by the pathway, wherein as some pathway products increase, the material that is allotted to others decreases. The state‐dependency of these trade‐offs demonstrates the emergence of epistasis from the simple kinetic model.

## Discussion

### COLOR TRANSITIONS OCCUR VIA CHANGES AT A SUBSET OF PATHWAY LOCI

In simulations of pathway evolution, we largely recover the same hotspot loci as seen in nature. For example, transitions from blue delphinidin to red pelargonidin production in *Iochroma* and *Penstemon* (Smith and Rausher 2011; Smith et al. 2013; Wessinger and Rausher 2014, 2015), blue delphinidin to purple cyanidin in *Phlox* (Hopkins and Rausher 2011), and purple cyanidin to red pelargonidin in *Ipomoea* (Marais and Rausher 2010) were accomplished by mutations at F3'5'H, F3'H, and DFR. In fact, these three loci, particularly F3'H and F3'5'H, are consistently the targets of fixed mutations underlying color variation in nature (reviewed in Wheeler and Smith 2019; see also Table S1). Our simulations clearly demonstrate that the hotspot nature of these enzymes is derived from their position in the pathway structure, which imparts a relatively higher ability to control flux. In other words, these are the loci that are able to perturb the pigmentation phenotype sufficiently to be selected on. This observation is in line with empirical findings in other metabolic pathways, such as those responsible for carotenoid biosynthesis in plants, wherein pathway position strongly affects the rate of fixed mutations at pathway loci (Clotault et al. 2012b). The overall effect of this narrowing of mutational targets is to impart some predictability and repeatability to the evolution of the pathway, particularly at the scale of which enzymes are likely to be involved. However, the dependence of mutational effects on the selected phenotypic transition demonstrates the need for knowledge of the precise context in which the pathway is evolving in order to make accurate predictions. For example, in cases of parallel evolution of a new pigmentation phenotype from a known common ancestral state, it will likely be possible to predict the targets and types of mutations (Wessinger and Rausher 2012).

Further consistent with our simulated results, natural transitions in anthocyanin pigmentation are often accomplished with a small number of functional changes, although these may be composed of several mutational steps at the genetic level. For example, the blue‐to‐red transition in *Iochroma* was achieved by the combined effect of three changes (deactivation of F3'5'H, down‐regulation of *F3'h*, and coding mutations in DFR) (Smith and Rausher 2011), while the *Phlox* transition required two (coding mutations at *F3'5'h* and a Myb transcription factor that regulates *F3'5'h* expression). Meanwhile, the *Penstemon* transition required only one change (deactivation of F3'5'H) as did the *Ipomoea* transition (down‐regulation of *F3'h*) (Marais and Rausher 2010). The large‐effect mutations at F3'5'H and F3'H in these empirical cases were in line with the results of our model, where we saw shifts of several‐fold in the $K_{cat}$, $K_{m}$, and $E_{t}$ parameters that resulted in large changes to activity of these enzymes. Similarly small numbers of fixed changes have also been observed in a variety of other evolutionary transitions, such as changes in floral morphology in *Mimulus* (Bradshaw et al. 1998), selection for growth rate in *Aspergillus nidulans* (Schoustra et al. 2009), adaptation to nutrient‐limitation in *Saccharomyces cerevisiae*, and adaptive melanism in *Chaetodipus* mice (Nachman et al. 2003; Steiner et al. 2007). This pattern reinforces the notion that major transitions can have a relatively simple genetic basis and can thus happen on short timescales given a sufficiently high rate for the generation of genetic variation (Gervasi and Schiestl 2017).

One notable difference between the set of hotspot enzymes so far known in nature and that of our simulations is the flavonol synthase (FLS) enzyme, a common target of fixed mutations in the pathway model. FLS is the enzyme responsible for the synthesis of the flavonol compounds (kaempferol, quercetin, and myricetin) from the DHK, DHQ, and DHM precursors that are shared with the anthocyanin branches (Fig. 1). Flavonols play a variety of critical biological roles including acting as sunscreen compounds that shield tissues from UV radiation (Ryan et al. 1998, 2002a). Our modeling shows that mutations at FLS can alter anthocyanin pigmentation by increasing activity on the shared precursors and thus drawing flux away from the anthocyanin branches. Changes in FLS concentration can yield differential effects depending on the status of the other enzymes catalyzing reactions that lead down any particular anthocyanin path. This result highlights the fact that, because of interactions among loci in the pathway, even those enzymes not required for anthocyanin biosynthesis can affect pigmentation. To our knowledge, only one empirical study of flower color variation has identified FLS as a target. In this case, differential regulation of FLS induces flower color pattern variation by tuning anthocyanin concentration rather than causing a shift between pigments (Yuan et al. 2016). However, it is worth noting that many searches for color loci have focused on candidate genes required for anthocyanin biosynthesis, so FLS has not received much attention. Additionally, FLS has been targeted to induce a switch between two anthocyanin pigments in genetically‐engineered systems (Table S1) (Nielsen et al. 2002; Nakatsuka et al. 2007). Based on our simulated results, FLS can therefore be thought of as a hotspot for “missing mutations” that we may expect to observe often in nature, but so far have only observed very rarely.

Another pathway enzyme that is rarely, if ever, involved in color transitions in nature is ANS (Table S1). In contrast to FLS, this observation is easily rationalized by our model. ANS has very little differential control over flux down the anthocyanin branches, because it is at the very end of the pathway (Fig. 1; Fig. S12). By the time precursors reach ANS, the bulk of relative flux partitioning down the pathway branches has already been determined by the upstream F3'H, F3'5'H, and DFR; that partitioning is then relatively insensitive to changes at ANS. Nevertheless, ANS was a more common target of fixed mutations when selecting on increased absolute concentration in the simple linear pathway control (see Methods; Fig. S10), consistent with the ability of ANS to potentially act as a final “bottleneck” for overall flux of pathway material toward anthocyanin production. These observations are in line with the occasional involvement of ANS as a target in transitions between pigmented and un‐pigmented phenotypes in flowers (Keiichi Shimizu 2011) and fruits (Rafique et al. 2016).

### RANGE OF TARGETS POINTS TO PLEIOTROPIC EFFECTS OF BIOCHEMICAL MUTATIONS IN NATURE

While we largely recovered the same hotspot loci as those observed in nature, the types of mutations in our simulations are broader. Specifically, although regulatory mutations (represented by enzyme concentration in our model) were preferred at all four hotspot loci, we also observed a substantial fraction of fixed biochemical mutations (those changing kinetic enzyme parameters). This contrasts with what is so far known about pigmentation transitions in nature, wherein changes at some loci are predominantly regulatory while changes at others tend to be structural (Wessinger and Rausher 2012). *F3'h*, in particular, has been primarily targeted by regulatory mutations in nature (Table S1), although it has also occasionally been targeted by biochemical activity‐tuning modifications (Hoshino et al. 2003; Zufall and Rausher 2003). In contrast, direct modifications of enzyme activity (in particular the alteration of specificity for anthocyanidin precursors) appear to be much more common than regulatory mutations at DFR (Table S1). This may be due to the need for modifications that alter DFR activity to tune pathway flux after shifts induced by regulatory mutations at branching enzymes, which redirect flux down the target branch (Zufall and Rausher 2004; Smith and Rausher 2011; Smith et al. 2013).

This discrepancy in the distribution of mutation types between our model and empirical observations may be explained by pleiotropic effects that occur in nature, particularly for mutations that affect enzyme function. Most of the enzymes in the anthocyanin pathway play multiple roles yet are encoded by single‐copy‐number genes (Winkel‐Shirley 2001). Therefore, changes in the enzymatic activity at the protein level would affect the enzymes in all tissues where they are expressed. If the change in activity induced by the mutation is detrimental to other functions when occurring in some tissues, this would impose a constraint on the allowable mutations (Wagner and Zhang 2011). In contrast, a tissue‐specific regulatory mutation could avoid this antagonistic pleiotropy by allowing activity in the off‐target tissue to remain unchanged (Streisfeld and Rausher 2011; Streisfeld et al. 2011; Wessinger and Rausher 2012). As an example, in the case of simulated transitions from cyanidin production to pelargonidin production, we observe changes that worsen effectiveness of F3'H on DHQ, the precursor for both leucocyanidin and quercetin. This will have the side effect of lowering quercetin production, and since quercetin is a more effective sunscreen than the monohydroxylated kaempferol produced from DHK (Ryan et al. 1998, 2002b), we would expect negative fitness effects from these changes. Alternatively, expression of *F3'h* is known to be under the control of different transcription factors in different tissues (Streisfeld and Rausher 2009; Smith and Rausher 2011), setting up the potential availability of tissue‐specific regulatory mutations that can alter the function while avoiding antagonistic pleiotropy. In contrast to *F3'h*, *F3'5'h* is thought to be a less pleiotropic gene overall, consistent with the predominance of fixed loss‐of‐function mutations at this locus (Wessinger and Rausher 2014, 2015) (Table S1).

Although we believe that pleiotropy explains many of the differences between our model and nature, aspects of our simulation design may also play a role. Our simulations treat the distribution of mutations equally for all genes and all regulatory and biochemical parameters, because the natural distributions are not known. Spontaneous color mutants in horticultural varieties provide a rough approximation of the mutation spectrum in terms of the relative number of regulatory and structural mutations (Streisfeld and Rausher 2011), but are too few to define distributions of mutational effects. We also have detailed studies of the effects of fixed genetic differences between populations and species (Table S1), but these differences could result from a stepwise accumulation of smaller mutations. Future versions of our model could test the effects of mutational spectra that differ across pathway enzymes, such as would occur in the case of multiple loci controlled by a shared regulatory factor or constraints imposed by biophysical limitations on enzyme activity. We predict that such differences in the mutational spectrum would not qualitatively alter the principal targets of evolution or the sign of mutations at those enzymes, but would likely quantitatively change the relative proportions of fixed mutations at certain loci and the ratio of fixed biochemical to regulatory mutations.

### EVOLUTIONARY WALKS BETWEEN PHENOTYPES AVOID LARGEST STEPS FIRST

Our simulations suggest that although color shifts can occur with a few mutations, those with largest effect (in terms of selection coefficient) do not tend to get fixed first. With a median of four selected mutations to reach the target phenotype, the largest mutations are typically fixed at the second step. We demonstrated that this result is not driven by the overall distribution of selection coefficients for modeled mutations, which span a wide range of values and follow a roughly exponential distribution (Fig. S7). Furthermore, the underlying distribution of parameter effect sizes for the sampled mutations is constant across all steps of the trajectories and the form of this distribution does not qualitatively affect the results. Our finding that the largest steps are not fixed first runs counter to some well‐established theoretical results for adaptive walks, which predict an exponential distribution of mutation sizes over trajectory steps (Orr 1998; Rokyta et al. 2005; Joyce et al. 2008; Orr 2002). However, it is worth noting that these studies have several limitations including the assumption that all the mutations are drawn from the same unchanging distribution of fitness effects and involve a single locus (Orr 1998; Joyce et al. 2008). We suspect that the deviation of our model from the theoretical prediction may be due in part to the violation of the typical assumptions, in particular that the branched pathway contains complex interactions between loci. As described in the Results, evolution of a simplified linear pathway did in fact yield a roughly exponential distribution of fixed selection coefficients. However, it appears that the constraints imposed by the fitness function used in our model (directional toward a specific target‐pigment ratio optimum and stabilizing on total steady state concentration) had a larger effect on the distribution. We argue that, under this fitness function, very large‐effect mutations that move the pathway phenotype in the correct direction are difficult to achieve, and thus rare, at the beginning of trajectories. In contrast, it appears that moderately‐sized mutations can disrupt the system sufficiently to open up new possible paths for subsequent mutations to facilitate further movement toward the optimum.

One of the key observations of this study is that the concept of “large effect” versus “small effect” mutations is not fixed for a change of a given size at a given parameter. The effect size of any given parameter mutation on the pathway fitness is instead contingent on the location of the phenotypic optimum and the distance of the pathway phenotype from the optimum at each step of a trajectory. Thus, the distribution of mutational effects on fitness shifts as the pathway evolves, rather than remaining static. We suggest that these dynamics are likely to be common for phenotypes controlled by intertwined networks of genes or enzymes, where epistasis is expected to be pervasive. Interestingly, a similar adaptive curve to those observed in this study (sigmoidal in the fitness space) emerges from a simple biophysical model of transcription‐factor network evolution (Lässig 2007) and peaked distributions can also be observed in models of evolution with shifting optima (Collins et al. 2007). Furthermore, under certain extreme value domains fitness effects are expected to increase, rather than decrease, over the course of adaptive walks (Seetharaman and Jain 2014).

It is difficult to determine how well the observed order of effect sizes for fixed changes align with nature. Studies of the evolution of polygenic phenotypes in plants that have been done, using approaches such as QTL mapping, yield all of the differences between evolutionary end states, but the order in which they accumulated remains unknown (Bradshaw et al. 1998; Hodges et al. 2002). Furthermore, the effects of these mapped regions are likely to be different in any extant proxy organism than they would have been in the ancestral organism due to evolutionary separation and the accompanying effects of genetic background (Leips and Mackay 2000; Lunzer et al. 2010). Nonetheless, we do expect mutations with a range of effects on the phenotype, some large and some smaller. In fact, this has been observed in at least one natural flower color transition; blue to red flowers in *Iochroma* (where the change at *F3'h* has the largest effect, followed by that at *F3'5'h*, and finally by the change at DFR) (Smith and Rausher 2011).

## Conclusions

Here, we used a computational model of the anthocyanin pigmentation pathway to simulate evolution between a series of floral pigmentation phenotypes. Assuming a similar mutational process across all pathway enzymes, we found that the fixed mutations in simulated evolutionary trajectories are contingent on the current phenotype, the location of the optimum phenotype in pigmentation space, and the structure of the pathway. Our approach can be used as a general framework to study how the topology of genetic pathways interacts with selection to determine evolutionary trajectories. We could also envision expansion to a fully population genetic model in order to explore the effects of population dynamics (e.g., bottlenecks) on the distribution of selected mutations (Dittmar et al. 2016). One of the most fruitful avenues for future work would be to construct a more realistic model of the regulatory architecture, directly linking the regulation of pathway enzymes through shared transcriptional activators and repressors. This would allow the relative importance of cis‐ and trans‐regulatory mutations to be assessed, along with their effects on the emergence of phenomena such as antagonistic pleiotropy and sign epistasis.

## DATA ARCHIVING

The python library *enzo*, for running evolutionary simulations, is available on github (https://github.com/lcwheeler/enzo). The scripts used to run the simulations, Jupyter notebooks and scripts for the subsequent analyses, and the raw simulated datasets are available in an OSF online repository (https://osf.io/kxr23/). Additional information is available in the supplemental text.

## AUTHOR CONTRIBUTIONS

L.C.W. and S.D.S. conceived the study and outlined the computational approach. L.C.W. constructed the mathematical model, wrote the code, performed the simulations, conducted the data analysis, and generated figures. L.C.W., B.A.W., and S.D.S. collaborated on writing the manuscript. S.D.S., L.C.W., and B.A.W. secured funding for the work. All authors have read and approved the manuscript.

## CONFLICT OF INTEREST

The authors declare no conflicts of interest.

Associate Editor: K. Lythgoe

## Supporting information


**Fig S1**. Alternate diagrams of the anthocyanin pathway.
**Fig S2**. Distributions of simulated trajectory lengths.
**Fig S3**. Fixation events per evolvable model parameter.
**Fig S4**. Effects of DFR concentration changes on anthocyanin output.
**Fig S5**. General effects of kinetic differences between enzymes.
**Fig S6**. End‐state normalized directional shifts for all evolvable parameters.
**Fig S7**. Aggregated distributions of fixed selection coefficients.
**Fig S8**. A simple linear model exhibits monotonically‐decreasing selection coefficients over trajectory steps.
**Fig S9**. Altering the fitness function has a strong affect on the stepwise distribution of selection coefficients.
**Fig S10**. Altering the fitness function and pathway topology results in different path way hotspots.
**Fig S11**. Sensitivity values for each parameter calculated from selection coeffcients and normalized directional mutation sizes.
**Fig S12**. Matrices of concentration control coeffients for the median evolved states.
**Fig S13**. Distributions of relative CHS control for evolutionary end‐states.
**Table S1**. Empirical studies of the genetic basis for differences in floral anthocyanin type in natural and engineered systems.
